# Spondylolisthesis and Idiopathic Sarcopenia Treated With Minimally Invasive Surgery for Transforaminal Lumbar Interbody Fusion: A Case Study and Literature Review

**DOI:** 10.7759/cureus.25086

**Published:** 2022-05-17

**Authors:** Taha Khalilullah, Siri Tummala, Ripul Panchal

**Affiliations:** 1 Neurological Surgery, American Neurospine Institute, Plano, USA; 2 Medical Student, TCU School of Medicine, Fort Worth, USA

**Keywords:** thoraco-lumbar spine, minimal invasive approach, sarcopenia, lumbar spondylolisthesis, transforaminal lumbar interbody fusion, minimally invasive surgery, ­wound healing, lumbar spine surgery, degenerative disc disease

## Abstract

Sarcopenia is a muscle-wasting disease common among older adults. The condition has been associated with adverse perioperative and postoperative outcomes following spinal surgery. The combination of this muscle-wasting syndrome and spondylolisthesis and how we approached the case makes it a compelling study for surgeons attempting to treat this patient population more effectively. In this case study, we examine a 76-year-old male patient with chronic sarcopenia who needed transforaminal lumbar interbody fusion (TLIF) surgery for his grade 1 L4-5 spondylolisthesis, L4-5 degenerative disc disease, bilateral facet effusions and lumbosacral radiculopathy with active and chronic denervation. He consulted our neurosurgeon for his back pain and left lower extremity paresthesia. Magnetic resonance imaging (MRI) showed degenerative disc disease with bilateral facet effusion in multiple levels of the lumbar spine as well as broad disc bulge in L5-S1. Due to the patient’s past medical history of muscle wasting disease, a muscle biopsy of the left quadriceps was performed and revealed rare denervated fibers indicative of sarcopenia. Minimally invasive transforaminal lumbar interbody fusion (MIS TLIF) was performed as the most optimal surgical method for this condition. The patient experienced a massive decline in his VAS score from 9/10 to 0/10 two months from surgery, reflecting the fast wound healing process and recovery. Postoperatively, the AP X-ray of the lumbar spine showed dextroscoliosis and stable L4/5 TLIF instrumentation. The surgeon provided the patient guidance regarding his nutrition and exercise to maximize the treatment. This case illustrates the employment of the minimally invasive surgery (MIS) approach to diminish complications and tissue trauma of patients with sarcopenia and spondylolisthesis who are undergoing lumbar spine surgery.

## Introduction

Sarcopenia is recognized as a progressive skeletal muscle disease associated with low muscle strength and function, older age, and additional factors [1]. Advancements have been made regarding the standardized definition, cause, and diagnostic criteria for the disease. For instance, although sarcopenia has been associated with the elderly, it is now understood that the progression of the disorder begins earlier in life [2]. In addition, low muscle strength, muscle quality, and physical performance have become the primary methods of defining sarcopenia. As such, maintaining muscle strength and mass throughout adulthood is essential to delaying sarcopenia [3]. Other factors for sarcopenia include a lack of exercise, inflammatory-inducing conditions, acute injury, obesity, and malnutrition [4-6]. 

As a result of this condition, individuals have a higher risk of falling, developing mobility disorders, a lowered quality of life, and death [7-9]. In addition, the prevalence of sarcopenia has been linked to increased frailty, disability, and more significant morbidity following spinal surgery [10]. As such, diagnosing patients for the disease is essential to account for adverse outcomes. In order to identify the condition, the European Working Group on Sarcopenia in Older People (EWGSOP) graded sarcopenia into two categories: primary sarcopenia and secondary sarcopenia [1]. Primary sarcopenia is categorized by only having age-related causes. Secondary sarcopenia is defined by factors other than age-related issues such as lack of physical activity, disease, and inadequate nutrition. The EWGSOP2 has identified new subcategories of acute and chronic sarcopenia. Acute sarcopenia occurs for less than six months with an associated acute illness and injury. Contrastingly, chronic sarcopenia lasts longer than six months with persistent conditions and higher mortality. Common muscle locations for sarcopenia include the psoas muscle, paraspinal area, and the mid-thigh muscle. The latter measures total body muscle volume and alterations precisely [1]. The disorder has been associated with poor surgical outcomes after lumbar spine surgery. Nevertheless, the interaction of spine pathologies, frailty, and sarcopenia remains inconclusive [11]. 

Upon reviewing the literature, research into the most optimal method to treat a patient with spondylolisthesis and the degenerative syndrome has not been investigated extensively. We present an interesting case of a 76-year-old male patient with L4-5 grade 1 spondylolisthesis and dynamic instability and chronic sarcopenia based on his extended history with the disease. As such, the complexity of this report stems from the patient's diagnoses and the reasoning for how we approached the surgery via minimally invasive transforaminal lumbar interbody fusion (MIS TLIF) to reduce tissue trauma and improve wound healing. The successful methods employed may enhance our knowledge about treating patients with spondylolisthesis and sarcopenia. Clinical characteristics, pathological features, and surgical nuances will be discussed.

## Case presentation

**Figure 1:**
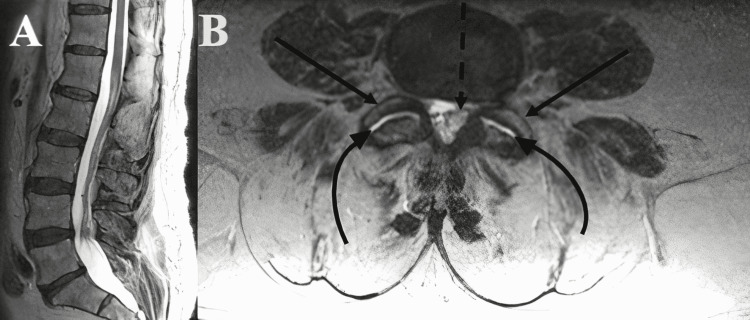
Preoperative MRI of the lumbar spine Sagittal image (A) and axial image (B) at L4/5 with bilateral facet arthropathy with facet effusion and left medial synovial cyst. MRI: magnetic resonance imaging; dashed arrow: left medial synovial cyst; curved arrow: bilateral facet effusion; straight arrow: bilateral facet arthropathy

**Figure 2:**
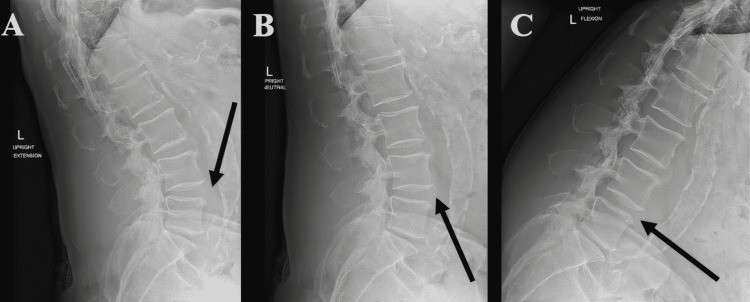
Preoperative X-ray of the lumbar spine Extension view (A) neutral view (B) and flexion view (C) of the lumbar spine with L4/5 grade I spondylolisthesis and diffuse spondylosis arrow: L4-L5 grade 1 spondylolisthesis

A 76-year-old male initially presented with symptoms of throbbing and radiating lower back pain with bilateral lower extremity paresthesia and ataxia. He had a medical history of obesity, hypertension, and smoking that he quit ten years ago. The patient previously attempted the following treatments: medication(s), physical therapy, and epidural steroid injections with no success. The patient has no pertinent surgical history. The patient had a work-up for his known family history of muscle wasting diseases identified eventually as sarcopenia. It was identified during the clinical examination that he had deep tendon reflex (DTR) 0+ for the patellar and Achilles bilaterally. In addition, the patient’s motor strength was 5/5 for the lower extremities. The patient’s sensation showed intact light touch and pinprick to lower extremities. Preoperative magnetic resonance imaging (MRI) of the lumbar spine revealed L2/3 degenerative disc disease, L3/4 degenerative disc disease with bilateral facet effusion, L4/5 degenerative disc disease with left facet synovial cyst with moderate to severe spinal stenosis with bilateral foraminal stenosis, and L5/S1 bilateral facet arthropathy as revealed in Figure 1A, 1B. The patient’s preoperative upright L-spine X-rays revealed L4/5 minimal grade I spondylolisthesis with diffuse spondylosis (Figure 2A-2C). 

**Figure 3:**
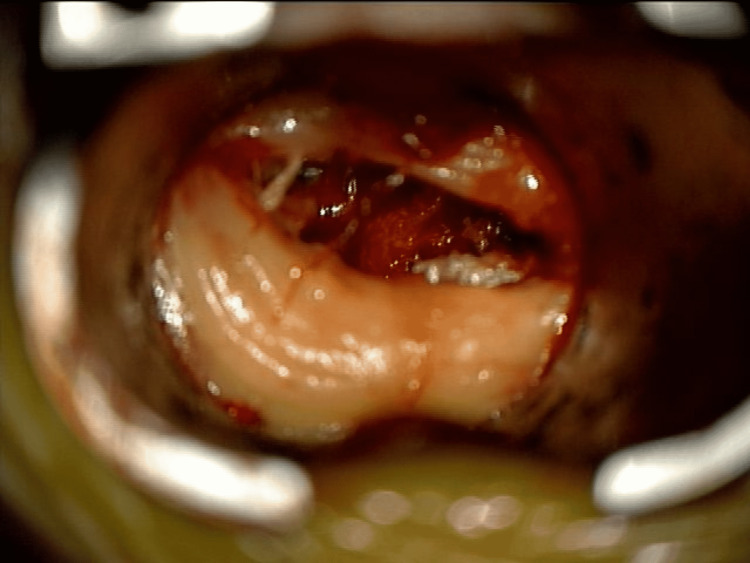
Intraoperative photograph showing a modified tubular retractor view revealing extensive fat infiltration into the paraspinal muscles

**Figure 4:**
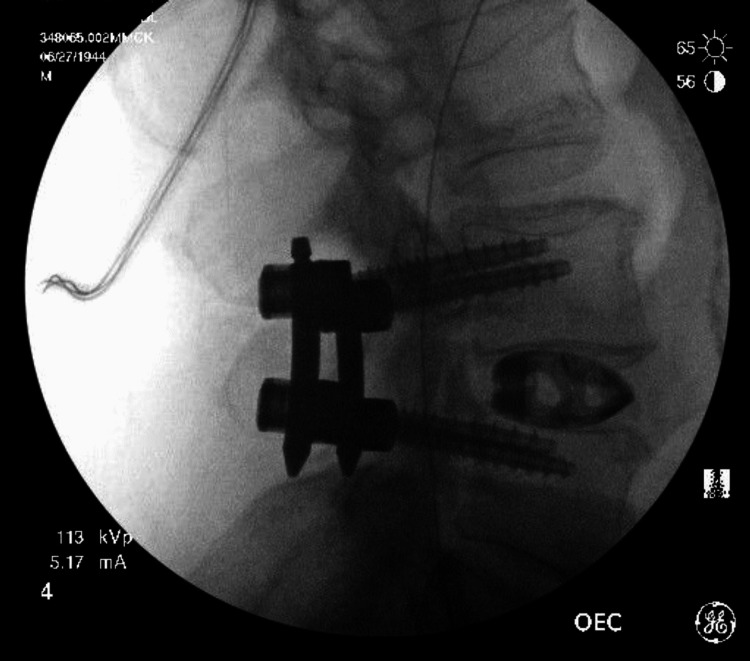
Intraoperative fluoroscopy image of L4-5 transforaminal interbody fusion stabilization

A muscle biopsy of the left vastus lateralis muscle in the left quadriceps showed acute/myopathic changes, angulated atrophic fibers, and rare denervated myofibers indicative of sarcopenia. In addition, intraoperative imaging revealed extensive fat infiltration into the paraspinal muscles (Figure 3). Upon examination, the patient was diagnosed with lumbar radiculopathy, dextroscoliosis, spinal stenosis, and sarcopenia. After shared decision-making, we proceeded with the MIS TLIF procedure. The patient was brought to the operation room, where fluoroscopy was employed to identify the area of interest. Local landmarks were utilized to insert a percutaneous intraoperative navigation reference frame on the patient’s posterior superior iliac crest. Then, the O-arm was used to acquire radiographic imaging, registration was completed on the image guidance system, and the accuracy of the navigating probe was confirmed using local landmarks. The midline and the bilateral incisions for insertion of both bilateral pedicle screws were identified. The left incision was planned to be used for the transforaminal approach. A linear incision in the bilateral paraspinal muscles was created. Then, the placement of pedicle screws on the right side with the initial incision through the fascia was made. On the contralateral incision, the placement of the screws was held off, but the pedicle screw hole was prepared. At this point, a tubular retractor system was employed for site expansion to expose the disc level at L4-5. Then, a high-speed drill and osteotomes helped complete the laminectomy on the left side and remove the facet joint, leading to local decompression. The synovial cyst was identified and was carefully dissected using microsurgical techniques. The thecal sac was retracted medially to expose the underlying disc space. The discectomy was completed in a piecemeal fashion. Endplates were decorticated for arthrodesis in the disc space and prefilled on the contralateral side with autograft. Next, an appropriate-sized cage was determined. Then, the 3D-printed titanium cage was filled with the patient’s autograft leading to the insertion of a 14 mm cage (Adaptix^TM^, Medtronic interbody system) placement with navigation. A good snug fit was noted. Ipsilateral screws were inserted and connected with rods and setscrews. Screws were stimulated prior to rod placement, and O-arm confirmed the adequate position of all implants. Intraoperative fluoroscopy image of L4-5 transforaminal interbody fusion revealed proper stabilization of the implant (Figure 4). Closure of the wound occurred in a standard manner. The estimated blood loss was 50ml.

**Figure 5:**
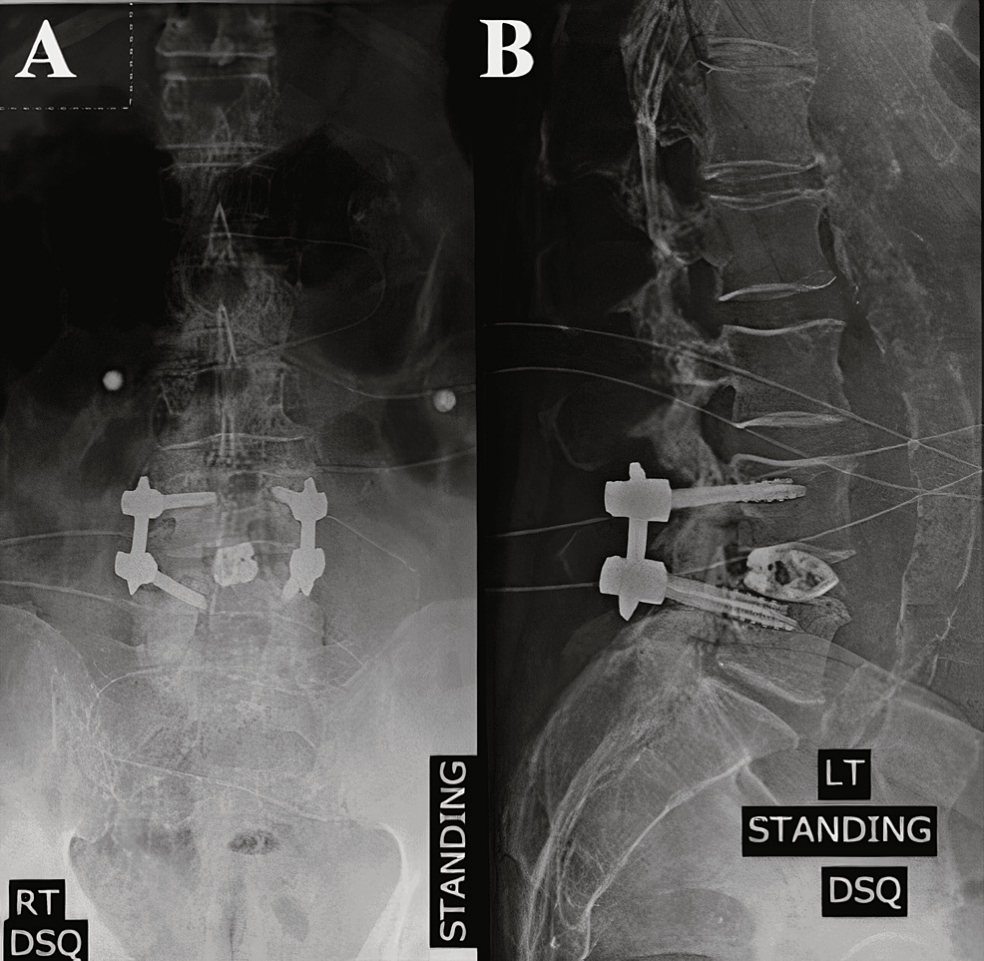
AP (A) and lateral (B) postoperative lumbar spine X-rays

After two months postoperatively, the back pain reduced from 9/10 to 0/10 on the VAS scale. However, the patient did report back pain without radiculopathy for a brief time one month after surgery. In addition, the L4/5 TLIF instrumentation was stabilized, as revealed by the 5-month postoperative AP and lateral X-ray (Figure 5A, 5B). The patient adhered to the interventions and tolerated them based on the interactions in the two-week, one-month, three-month, six-month, and one-year postoperative visits. No adverse or unexpected events arose, and the patient noted that the preoperative symptoms resolved after six months postoperatively. 

## Discussion

This study demonstrates the application of MIS TLIF to possibly minimize tissue trauma and improve postoperative outcomes for sarcopenia patients with spondylolisthesis. The case is notable because of the rationale as well as the approach in treating the patient who had L4-5 grade 1 spondylolisthesis with dynamic instability and sarcopenia. Consequently, it is essential to realize the relation between spondylolisthesis and sarcopenia. For background, in a cross-sectional design by Matsuo et al., the sarcopenia group presented lower levels of appendicular, lower trunk, and average muscle mass in comparison to the nonsarcopenic group. As a result, there was a significantly higher occurrence of slippage in the lumbar spine for sarcopenia patients with a direct correlation by multiple regression analysis [12]. In connection, a biomechanical study by Zhu et al. demonstrated that the reduced strength of the back muscles might intensify degenerative spondylolisthesis, reflecting the importance of trunk muscles in maintaining the lower spinal column [13]. With lumbar spine surgeries disrupting paraspinal muscles and destabilizing the axial skeleton to varying degrees, the associated muscles are further aggravated, possibly leading to advanced lumbar spondylolisthesis. In addition, patients with sarcopenia have been shown to have a decrease in the regeneration of skeletal muscles due to the impaired satellite cells impeding proper recovery for the muscles postoperatively [14]. Regardless, it is valuable to recognize that the research on sarcopenia treatment options and the impact of the syndrome on related surgeries are still in the initial stages of development. On one side, Bokshan SL et al. found that individuals with sarcopenia had worse cumulative survival, 1.7 times longer hospital stays, and three times greater complication rates than patients without sarcopenia after thoracolumbar spine surgery [15]. Thus, sarcopenia may be a pivotal factor to consider with spine surgery. Furthermore, Hirase et al. demonstrated that individuals with sarcopenia measured by psoas muscle index had more frequent perioperative adverse events, 30-day readmission rates, and 30-day reoperation rates than patients without sarcopenia during complex revision thoracolumbar spine surgery [16]. Also, the study by Inose et al. on the influence of sarcopenia on lumbar surgery concluded a lower recovery rate and inferior Japanese Orthopedic Association (JOA) score for the sarcopenia group [17]. As such, postoperative outcomes are not optimal with spine patients diagnosed with the disease. On the other hand, a report by McKenzie et al. revealed that the clinical prognosis and surgical complications in lumbar fusion surgery for patients with and without sarcopenia were remarkably similar. In addition, both groups reflected identical outcomes in the long term, demonstrating the inconsequential result of the condition [18]. Similarly, in a study by Toyoda et al., the researchers determined that the non-sarcopenic and sarcopenic patients who had minimally invasive lumbar decompression surgery presented clinical assessments such as JOA score, VAS for LBP, and leg numbness that were not significantly different. Additionally, the JOA score improved postoperatively regardless of a sarcopenic diagnosis [19]. Thus, the reports could indicate that it has little impact on minimally invasive procedures such as the one performed in this case study. The inconclusiveness concerning the results of sarcopenia on the clinical complications of spinal surgeries demonstrates the need for further examination regarding the influence of the muscle-wasting disease. Because postoperative outcomes remain a concern for sarcopenia patients, optimizing the procedure is essential for all surgeons to ensure the best prognosis. 

The main principle for treating spondylolisthesis in patients with a history of sarcopenia includes minimizing muscle atrophy and fat infiltration [20]. As such, our initial experience with such patients was that MIS is the best approach. Because the TLIF approach necessitates significant soft tissue and paraspinal muscle dissection and retraction to access the vertebral column, there can be worsening pain and prolonged recovery in patients undergoing this approach [21]. To avoid these detrimental outcomes, MIS TLIF can minimize tissue trauma, create more minor wounds, speed up the recovery process, decrease blood loss, and reduce surgical site infections [22]. Furthermore, in contrast to the posterior lumbar interbody fusion (PLIF) procedure, TLIF shows more improvements in both pain and various measures of quality of life, demonstrating the importance of MIS [23]. In the context of a patient with sarcopenia, MIS TLIF may help mitigate the progression of the condition. In relation, the results described a significant reduction in back pain over a six-month period post-surgery. The wound healing process occurred in one month with no related adverse complications, emphasizing the lack of tissue damage. Thus, MIS may have played a crucial role. However, other confounding factors such as exercise and vitamin D may have also contributed to an improved patient outcome and wound healing [18,24]. Regardless of the perceived short healing period, immense reduction in the VAS score, and other enhanced patient measurements in this report, no conclusions can be reached about MIS TLIF being a promising treatment method for patients with lumbar spondylolisthesis and sarcopenia. Further studies must be done to compare the contribution of MIS and other surgical techniques to assess the findings in this case and identify the best operative method. Additionally, prospective data with a more significant number of patients and standardized patient questionnaires like SARC-F for screening sarcopenia risk would reach a more effective conclusion.

## Conclusions

The most optimal surgical route to reduce tissue trauma and improve wound healing for patients with sarcopenia and lumbar spondylolisthesis has not been determined. Yet, in this case study, we found notable success by employing the minimally invasive transforaminal lumbar interbody fusion (MIS TLIF) procedure, resulting in a short healing period and a significantly improved patient outcome. Further examination is needed to determine a proper understanding. Nevertheless, this report may serve to spark such interest to either confirm, contradict, or find another solution that serves sarcopenia patients with spondylolisthesis, improving the results of this patient population.
